# Osmotic stress and melatonin in *Pfaffia glomerata*: biochemical responses and 20-hydroxyecdysone modulation

**DOI:** 10.1007/s00709-026-02193-y

**Published:** 2026-04-02

**Authors:** José Victor S. Silva, Tatiane D. Silva, Sérgio Heitor S. Felipe, Juliane M. Henschel, Diego S. Batista, Evandro A. Fortini, Letícia M. Farias, Michelle Maylla V. Almeida, João Paulo V. Leite, Kleiton L. G. Machado, Wagner C. Otoni

**Affiliations:** 1 Departamento de Biologia Vegetal/Laboratório de Cultura de Tecidos Vegetais/BIOAGRO, Campus Universitário, Universidade Federal de Viçosa, Viçosa, MG 36570-900 Brazil; 2 Universidade Federal Rural da Amazônia, Capitão Poço, PA 68650-000 Brazil; 3 Programa de Pós-graduação em Agronomia, Universidade Federal da Paraíba, Areia, PB 58397-000 Brazil; 4 Departamento de Bioquímica e Biologia Molecular, Laboratório de Biodiversidade, Universidade Federal de Viçosa, Viçosa, MG 36570-900 Brazil; 5 Departamento de Botânica e Zoologia, Universidade Federal do Rio Grande do Norte, Natal, RN 59078-970 Brasil

**Keywords:** Antioxidant defense, Brazilian-ginseng, Medicinal plant, Membrane damage, Osmolytes

## Abstract

Osmotic stress, driven by factors such as soil drying and salinization, poses a significant challenge to plant growth and metabolism. Melatonin has shown promising results in alleviating abiotic stress by inducing antioxidant defenses in several species, which is particularly interesting when considering medicinal plants. Thus, this study investigates the physiological and biochemical responses of Brazilian-ginseng (*Pfaffia glomerata*) to PEG 4000-induced osmotic stress and the mitigating effects of exogenous melatonin. Under osmotic stress, *P. glomerata* exhibited reduced growth, diminished photosynthetic pigments, and increased reactive oxygen species (ROS) levels, highlighting the detrimental impact of water deficit on plant health. Melatonin, in turn, differentially affected leaves and roots, failing to restore shoot growth while promoting root elongation under osmotic stress. In addition, melatonin increased the activity of antioxidant enzymes, particularly peroxidase (POD), reducing ROS production and membrane damage in the leaves. In roots, PEG only increased catalase (CAT) activity. Osmotic adjustment following melatonin application was also evident through elevated sucrose and proline levels, supporting cell turgor and stress adaptation. Interestingly, osmotic stress increased 20-hydroxyecdysone (20-E) levels in roots; however, this increase occurred independently of melatonin. Therefore, despite the induction of osmotic adjustments and antioxidant defenses, melatonin was unable to reverse the growth restraints caused by osmotic stress in *P. glomerata*. Furthermore, our findings reveal a complex interplay between osmotic stress, antioxidant defenses, and secondary metabolite production. The insights gained offer potential applications for improving stress resilience and secondary metabolite synthesis in medicinal plants, with implications for sustainable agriculture.

## Introduction

Water is essential for plant life, and its movement from soil to atmosphere is driven by gradients in water potential along the soil–plant–atmosphere continuum (Haberstroh et al. 2025). Drought and salinization may concentrate solutes in soil solution, decreasing water potential and resulting in osmotic stress, which disrupts the water potential gradient and, thus, prevents water uptake by plants (Wang et al. 2023; Yu et al. 2024). As we enter the “global boiling era”, climate changes are driving to more frequent and intense drought events, increasing the threat of osmotic stress to agricultural production (Amnuaylojaroen 2023). Drought causes plant dehydration, reducing photosynthetic efficiency and growth, also inducing oxidative stress through the overproduction of reactive oxygen species (ROS) (Sato et al. 2024). In addition to reduced water uptake, salinity also leads to ion toxicity through to the accumulation of Na^+^ and Cl^−^ in plant tissues, directly impairing plant physiology, and ultimately leading to plant death (Zhou et al. 2024).

Osmotic stress is a physical phenomenon that affects cell turgor and volume, cell membrane tension, and cell wall stiffness and integrity (Yu et al. 2024). Plants primarily detect osmotic changes through osmosensors, which monitor plasma membrane properties such as turgor and tension (Haswell and Verslues 2015; Nongpiur et al. 2020). Changes in cell turgor also lead to macromolecular crowding, a phenomenon in which the cytoplasm volume is reduced, affecting cellular physiology (Meneses-Reyes et al. 2024). Plants cope with osmotic stress through different strategies, from the molecular to the tissue level. For instance, they can modulate root growth through hydrotropism and halotropism, changing the direction of growth to explore root surroundings better reaching more water or escaping from areas with high salt concentration (Miyazawa and Takahashi 2020). They also modulate stomatal aperture and cuticular composition, as well as induce early formation of casparian strip and suberin lamelae to reduce water loss (Hasanuzzaman et al. 2023; Kim and Sung 2023). Moreover, the accumulation of compatible solutes in cells, such as polyamines, betaines, sugars, amino acids and sugar alcohols, reduces cell water potential, maintaining hydration and preventing desiccation (Theillet et al. 2014; Kaur et al. 2024). Other responses to osmotic stress include modulation of ROS production and signalling, as well as aquaporins the localization in cell membranes (Yu et al. 2024).

In vitro plant tissue culture is widely used to investigate osmotic stress under controlled conditions. This approach allows for precise control of environmental factors such as light (Silva et al. 2021), gas concentrations (e.g., CO_2_) (Saldanha et al. 2012), and secondary metabolite production in response to stress (Martínez-Santos et al. 2021). Moreover, Polyethylene glycol (PEG) is a common osmotic stress agent, as demonstrated in studies with barley (Kocheva and Georgiev 2003), sorghum (O’Donnell et al. 2013), alfalfa (Zhang and Shi 2018), the medicinal plant *Thymus vulgaris* (Razavizadeh et al. 2019), and even halophytic species like *Sesuvium portulacastrum* (Slama et al. 2007). This system is also suitable for testing protective molecules such as melatonin.

Melatonin (N-acetyl-5-methoxytryptamine) is an indoleamine synthesized by plants, and exhibits pleiotropic effects similar to those of hormones (Ludwig-Müller and Lüthen 2015; Cai et al. 2025). These effects include regulating ROS production and signaling (Wei et al. 2018), controlling membrane permeability and intracellular calcium levels (Li et al. 2016), and modulating osmoregulatory molecules such as sugars and branched-chain amino acids (Shi et al. 2015; Wei et al. 2015). Under salinity, melatonin enhances photosynthetic performance and membrane integrity and reduces H₂O₂ accumulation, but does not consistently decrease MDA, indicating protective effects largely independent of membrane glycerolipid remodeling (Ayaz et al. 2026). Additional roles of melatonin have been extensively reviewed in previous studies (Arnao and Hernández-Ruiz 2019; Luo et al. 2025). These properties make melatonin a promising candidate for improving abiotic stress tolerance and enhancing secondary metabolite production. For instance, melatonin increased both stress tolerance and secondary metabolite accumulation in tomato plants exposed to high nickel levels (Jahan et al. 2020), as well as in rosemary (*Salvia rosmarinus*) and citrus during drought and arsenic stress (Farouk and Al-Amri 2019; Jafari and Shahsavar 2021). These findings suggest its potential application across diverse species, including *Pfaffia glomerata*.

Plant melatonin does not act in isolation but interacts with multiple hormonal signaling pathways that regulate plant growth and stress responses. Increasing evidence indicates that melatonin participates in crosstalk with classical phytohormones such as abscisic acid (ABA), auxin, brassinosteroids, and ethylene. Under drought and osmotic stress, melatonin has been shown to interact with ABA-dependent signaling pathways involved in stomatal regulation, antioxidant activation, and osmotic adjustment (Arnao and Hernández-Ruiz 2019; Luo et al. 2025). In addition, melatonin shares biosynthetic and functional similarities with auxin and can influence root growth and architecture through auxin-related pathways (Ludwig-Müller and Lüthen 2015). Crosstalk between melatonin and brassinosteroid signaling has also been proposed, particularly in the regulation of stress tolerance and redox homeostasis (Bartwal et al. 2013; Manghwar et al. 2022). Furthermore, melatonin may influence ethylene-related stress responses that regulate growth inhibition and stress acclimation under drought and osmotic stress. Through these hormonal interactions, melatonin regulates redox balance, osmotic adjustment, and developmental responses under abiotic stress.

The Brazilian-ginseng [*Pfaffia glomerata* (Spreng.) Pedersen (Amaranthaceae)] is a medicinal plant traditionally used by indigenous South Americans as antidiabetic, tonic, aphrodisiac, anti-inflammatory, and analgesic (Ribeiro et al. 2024). These medicinal features are attributed to a broad array of bioactive compounds (Shiobara et al. 1993; Caleffi et al. 2015; Franco et al. 2021; Dinan et al. 2021). Among these compounds, 20-hydroxyecdysone (20-E) stands out for its similarity to the insect molting hormone ecdysone, and may play a role in plant defense against herbivorous insects (Dinan et al. 2009). Under non-stress conditions, *Pfaffia glomerata* accumulates 20-hydroxyecdysone at stable, organ-specific basal levels, typically ranging from ~ 0.42–0.66% of dry mass in roots and ~ 0.21–0.60% in leaves, with variation mainly associated with plant development rather than stress (Festucci-Buselli et al. 2008).

The 20-E may protect against plasma membrane damage caused by abiotic stresses, including osmotic stress (Li et al. 2018), lead contamination (Lamhamdi et al. 2016), and high irradiance (Silva et al. 2021), possibly through crosstalk with other plant hormones and antioxidant defense mechanisms (Arif et al. 2022). In a recent study, increased salinity (NaCl) in the growth medium led to a 47% rise in 20-E production in *P. glomerata* cultured in vitro (Felipe et al. 2019).

Based on these findings, we hypothesize that, under PEG-induced osmotic stress, melatonin modulates specific biochemical and metabolic components of the stress response in *Pfaffia glomerata*, including antioxidant enzyme activity, membrane integrity, osmotic adjustment, and tissue-specific 20-hydroxyecdysone accumulation, without necessarily restoring growth.

## Materials and methods

### Plant material and *in vitro* cultivation

*Pfaffia glomerata* plants were obtained from the in vitro germplasm bank of the Plant Tissue Culture Laboratory (LCT) at the Institute of Biotechnology Applied to Agriculture (BIOAGRO), Federal University of Viçosa (UFV), MG, Brazil. Voucher material was deposited at the Leopoldo Krieger Herbarium (UFJF, Juiz de Fora, MG, Brazil) under code number CESJ 63,317. Access to genetic material (permission number AA2A367) was granted by the Brazilian National System for the Management of Genetic Heritage and Associated Traditional Knowledge (SISGEN).

Nodal segments approximately 2 cm in length from 30-day-old *P. glomerata* (accession 43) were selected and transferred to glass flasks containing MS medium with vitamins (Murashige and Skoog 1962; Powder mixture M519, Phytotech Labs), without casein hydrolysate or growth regulators, and supplemented with 3% (w/v) sucrose. The medium was solidified with 0.55% (w/v) micropropagation-grade agar (Agar Plant TC, Phytotech Labs). The flasks were sealed with rigid polypropylene caps, each containing two 10 mm holes covered by hydrophobic fluoropore membranes (PTFE, MilliSeal AVS-045 Air Vent) with 0.45 μm pores. The flasks were maintained for 40 days in a growth room under controlled conditions: a 16-h photoperiod, a temperature of 25 ± 2 °C, and an irradiance of 60 µmol m⁻² s⁻¹ provided by two LED lamps (SMD 100, 18 W, Vilux^®^, Vitória, ES, Brazil).

The treatments consisted of: (I) control; (II) melatonin; (III) polyethylene glycol 4000 (PEG); and (IV) melatonin + PEG. To induce osmotic stress, polyethylene glycol 4000 (PEG 4000; CAS 25322-68-3; analytical grade, Vetec Química Fina Ltda., Rio de Janeiro, Brazil) was added to the culture medium at a concentration of 2.5 g L⁻¹, while melatonin was applied at 100 µM (Sigma-Aldrich Co., St. Louis, MO, USA). The melatonin concentration was selected based on previous in vitro dose–response evidence indicating beneficial effects at low to moderate levels (100 µM) and growth inhibition at higher doses (≥ 500 µM) in *P. glomerata* (Silva et al. 2025), whereas the PEG concentration was chosen to induce moderate osmotic stress without causing lethality, allowing the evaluation of metabolic adjustment rather than collapse. Melatonin and PEG were applied through the culture medium on the tenth day of cultivation and maintained until the end of the 40-day cultivation period, characterizing a root-mediated treatment. The treatments were applied on the tenth day of cultivation to allow initial explant establishment and root development before stress imposition, ensuring that the responses reflected osmotic stress effects rather than early in vitro establishment. The plants were maintained in a growth room under a 16-h photoperiod, at 25 ± 2 °C and an irradiance of 60 µmol m⁻² s⁻¹ provided by two LED lamps (SMD 100, 18 W, Vilux^®^, Vitória, ES, Brazil).

The experimental design was entirely randomized, with four cultivation conditions: (I) control; (II) melatonin; (III) PEG; and (IV) melatonin + PEG. Each treatment had ten replicates, with the experimental unit consisting of a flask containing three explants.

### Biometric evaluation

The following growth variables were assessed: dry mass (g) of leaves, stems, and roots; stem length (cm), measured from the base shootwards; root length (cm), corresponding to the length of the longest root; number of leaves; and leaf area (cm²). To determine leaf area, leaves were photographed with a digital camera, and the images were processed using the ImageJ software (Schneider et al. 2012). The fresh plant material was dried in an oven at 50 °C for 72 h to obtain the dry mass.

### Photosynthetic pigments and anthocyanin determination

To determine the photosynthetic pigment content, 20 mg of fresh leaf material was combined with 700 µL of 100% methanol and incubated for 20 min at 80 °C in 96-well microplates. Absorbance readings were taken using a microplate reader (OptiMax Tunable Microplate Reader) at wavelengths of 470, 666 and 653 nm, which correspond to carotenoids, chlorophyll *a* (Chl *a*) and chlorophyll *b* (Chl *b*), respectively. Calculations were based on the formulas described by Wellburn (1994). The anthocyanin content was determined using the method proposed by Neff and Chory (1998). Approximately 10 mg of ground and lyophilized leaf material was mixed with 300 µL of acidified methanol (1% HCl w/v) and incubated overnight with shaking at room temperature. To separate the chlorophyll phase from the anthocyanins, 200 µL of distilled water and 500 µL of chloroform were added, and the solution was vortexed and centrifuged at 14,000 × *g* for 5 min. For plate assembly, 200 µL of the upper phase of the solution was collected. Absorbance readings were then taken at 530 nm (A₅₃₀) and 657 nm (A₆₅₇) using a spectrophotometer. The relative anthocyanin content was calculated by subtracting A₆₅₇ from A₅₃₀.

### Carbohydrates, amino acids, and protein quantification

Leaf and root samples were collected and subjected to methanolic extraction (Lisec et al. 2006). Approximately 10 mg of plant material, frozen in liquid nitrogen, grounded, and lyophilized, was sampled in microtubes. Subsequently, 700 µL of methanol was added to the samples, and the tubes were placed in a hot water bath. The samples were homogenized and incubated at 80 °C with shaking at 400 × *g* for 20 min, followed by centrifugation at 12,000 × *g* for 15 min at 4 °C. The supernatant was transferred to new microtubes, and a 150 µL sample was set aside to measure photosynthetic pigments. Then, 375 µL of chloroform and 750 µL of ultrapure water were added to the remaining supernatant. After thorough homogenization and centrifugation at 12,000 × *g* at 4 °C for 10 min, the upper clear aqueous phase was transferred to new microtubes and used to determine sugars and amino acids (Baxter et al. 2003; Sun et al. 2006). The precipitate from the methanolic extraction was washed twice with 70% ethanol and subsequently used to determine starch and protein content (Baxter et al. 2003; Panariello et al. 2017).

For sugar determination, 160 µL of the reaction mixture (100 mM HEPES/KOH with 3 mM MgCl₂, pH 7, 118 mM ATP, 48.4 mM NADP⁺, and 56 units of glucose-6-phosphate dehydrogenase (G6PDH, 5 mg mL⁻¹)) were added to each well of a 96 well microplate, followed by 25 µL of the methanolic extract and 25 µL of methanol. Absorbance readings were taken at 340 nm using a microplate reader, with intervals of 1 min between readings. After the optical density (OD) stabilized, 5 µL of each enzyme was added sequentially: hexokinase (1.5 U per reaction), phosphoglucose isomerase (0.7 U per reaction), and invertase (5 U per reaction), with approximately 30 min between each addition.

Amino acid content was determined by adding 30 µL of the methanolic extract, 20 µL of 70% ethanol, 50 µL of citrate buffer (pH 5.2) with 0.2% (w/v) ascorbic acid, and 100 µL of 1% (w/v) ninhydrin in 70% (v/v) ethanol to each well of the microplate. The microplate was incubated at 95 °C in the dark for 20 min, and absorbance was then read at 570 nm. A calibration curve was prepared using leucine as the standard.

Protein content was determined by resuspending the precipitate from methanolic extraction in 400 µL of 0.1 M NaOH. The microtubes were homogenized and incubated at 95 °C with shaking at 400 × *g* for 1 h, followed by centrifugation at 12,000 × *g* at 4 °C for 10 min. A 5 µL aliquot of the supernatant was added to a microplate containing 180 µL of Bradford reagent (diluted 1:5) in each well. Absorbance was measured at 595 nm, and a calibration curve was created using bovine serum albumin (BSA) as the standard.

Starch content was determined by adding 70 µL of 1 M acetic acid to the tubes containing the precipitate from methanolic extraction and 0.1 M NaOH to condition the pH for the reaction. A 40 µL aliquot of the suspension was transferred to a microplate containing 60 µL of a starch hydrolysis mix (comprising amyloglucosidase (0.14 units µL⁻¹) and α-amylase (0.01 U µL⁻¹)). The microplate was sealed with aluminum tape (3 M Model 425^®^) and incubated overnight at 37 °C. The hydrolyzed extract (25 µL) was transferred to a new microplate containing 25 µL of methanol and 160 µL of the reaction mixture (1 M HEPES/KOH buffer, pH 7.0, 3 mM MgCl₂, 118 mM ATP, 48.4 mM NADP⁺, and 56 units G6PDH (0.7 units µL⁻¹) per well). Absorbance readings were taken at 340 nm in a microplate reader, with 1-min intervals between readings. Once OD stabilized, 2 µL of hexokinase (2 units per reaction) was added to each well. Sugar and starch content was calculated using the Lambert-Beer equation: NADPH (µmol) = ∆_OD_/(2.85 × 6.22).

### Proline content

Proline content was determined following the methodology proposed by Bates et al. (1973). In a microplate, 50 µL of the methanolic extract was mixed with 100 µL of acidic ninhydrin solution (1% (w/v) ninhydrin in 60% (v/v) acetic acid and 20% (v/v) ethanol). The microplate was sealed with aluminum tape and incubated at 95 °C for 20 min. Absorbance was then measured at 520 nm, and a calibration curve was prepared using a standard proline solution (Sigma-Aldrich, St. Louis, MO, USA).

### Quantification of antioxidant enzymes

The enzymes catalase (CAT, EC 1.11.1.6), ascorbate peroxidase (APX, EC 1.11.1.11), superoxide dismutase (SOD, EC 1.15.1.1), and peroxidase oxidoreductase (POD, EC1.11.1.7) were quantified. 50 mg of fresh leaf and root material was homogenized with 1 mL of extraction medium (0.1 M potassium phosphate buffer, pH 6.8; 0.1 mM ethylenediaminetetraacetic acid (EDTA); 1 mM phenylmethylsulfonyl fluoride (PMSF) and 1% (w/v) PVPP). Centrifugation was carried out at 10,000 × *g* for 15 min at 4 °C, and the supernatant was used as a crude enzyme extract.

To determine CAT activity, 20 µL of the extract was combined with 200 µL of reaction medium (50 mM potassium phosphate buffer, pH 7.0 and 12.5 mM H_2_O_2_) in a microplate (Havir and McHale 1987). The absorption was measured at a wavelength of 240 nm for 1 min (every 10 s), and the enzyme activity was calculated based on the decrease in absorbance. For APX activity (Nakano and Asada 1981), the reaction medium (50 mM potassium phosphate buffer, pH 7.8, 0.25 mM ascorbic acid, 0.1 mM EDTA and 0.3 mM H_2_O_2_) was added to 20 µL of extract in the microplate and the absorbance was read at a wavelength of 290 nm for 1 min. POD activity was determined according to Maehly and Chance (1954). The reaction medium (13 mM guaiacol, 5 mM H_2_O_2,_ and 50 mM Na-phosphate pH 6.5) was added to the crude extract and read at 470 nm for 1 min. CAT, APX, and POD activities were expressed as µmol min^− 1^ mg^− 1^ protein.

SOD activity was determined according to the protocol described by Giannopolitis and Ries (1977), by adding 190 µL of reaction medium (50 mM sodium phosphate buffer, pH 7.8, 13 mM methionine, 75 µM p-nitro tetrazolium blue (NBT), 0.1 mM EDTA and 2 µM riboflavin) and 30 µL of crude extract to a microplate. The microplate with the samples was kept at 25 °C for 5 min in a reaction chamber (illuminated by a 15 W fluorescent lamp). After this time, the plate was removed from the light, and the reaction stopped. The photoreduction of blue tetrazolium increased the absorbance during the reaction, and the absorbance value was subtracted from the blank, which lacked enzyme extract. SOD activity was expressed as U min^− 1^ mg^− 1^ protein, with 1 U being equivalent to the concentration of SOD required to inhibit 50% of nitro blue tetrazolium photoreduction.

### Determination of lipid peroxidation and hydrogen peroxide levels

Lipid peroxidation was determined by quantifying the malondialdehyde (MDA) levels, using the methodology proposed by Heath and Packer (1968), with modifications. For the extraction, 500 µL of 1% trichloroacetic acid (TCA) was added to 50 mg of fresh, macerated plant material. The solution was vortexed and centrifuged at 12,000 × *g* for 15 min at 4 °C. Then 250 µL of the supernatant was transferred to new tubes and 750 µL of 0.5% 2-thiobarbituric acid (TBA; w/v) in 20% (w/v) TCA. The reaction was incubated by shaking at 95 °C and stopped in an ice bath after 30 min. The supernatants were transferred to new tubes and centrifuged (10,000 × *g*, 10 min, 4 °C), and read on a spectrophotometer at 532 (A532) and 600 (A600) nm. The concentration of MDA was given by subtracting A600 from A532, and calculated according to the extinction coefficient of 155 mM cm⁻^1^.

The hydrogen peroxide (H_2_O_2_) content was determined according to the methodology of Velikova et al. (2000). For the extraction, 500 µL of 0.1% trichloroacetic acid (TCA) was added to 50 mg of fresh, and grounded plant material. The solution was vortexed and centrifuged at 14,000 ×g for 15 min at 4 °C. In microplates, 20 µL of the supernatant, 80 µL of potassium phosphate buffer (10 mM and pH 7.0), and 100 µL of potassium iodide (1 M) were added. The reaction was incubated in the dark for 45 min and read using a spectrophotometer at 390 nm. The H_2_O_2_ content was calculated based on a standard curve.

### Total phenolics and flavonoids quantification

Phenolic compounds and total flavonoids were quantified using the Folin–Ciocalteu method (Tambe and Bhambar 2014). Briefly, 10 mg of macerated and lyophilized leaf tissue were subjected to methanolic extraction. For the total phenolic content determination, 25 µL of the extract was mixed with 25 µL of Folin–Ciocalteu reagent, 50 µL of 10% Na_2_CO_3_, and 100 µL of distilled water. The reaction was incubated in the dark for 2 h, and absorbance was measured at 760 nm using gallic acid as the standard.

For the quantification of total flavonoids, 20 µL of the extract was combined with 7.5 µL of 5% NaNO_2_, 30 µL of 2.5% AlCl_3_, 50 µL of 1 M NaOH, and 165 µL of distilled water. The absorbance was then measured at 500 nm, with catechin used as the reference standard.

### Quantification of 20-hydroxyecdysone

The methodology proposed by Kamada et al. (2009) was used to quantify 20-E in leaves. The methanolic extract was prepared as described by Côrrea et al. (2015) and quantification was carried out by high-performance liquid chromatography (HPLC) on a Shimadzu SPD-10Avp (Kyoto, Japan) apparatus equipped with an RP column (150 mm x 4.6 mm i.d., 5 μm particle size; C18 stationary phase) from Phenomenex (Torrance, CA, USA) and a Shimadzu SPD-M20A photodiode array detector (246 nm monitoring). The mobile phase consisted of ultrapure water (solvent A) and LC-grade methanol (solvent B). The gradient profile was: 0–8 min: 50% B; 8–13 min: 50–95% B. All analyses were carried out at a flow rate of 1.0 mL min^− 1^ at 40 °C. The volume of samples injected was 20 µL, with a duration of 15 min. The calibration curve was obtained by injecting 20-E standard (Sigma-Aldrich, St. Louis, MO, USA) into HPLC-grade methanol (0 to 120 mg L^− 1^). 20-E content leaves were expressed as mg g^− 1^ dry weight.

### Data analysis

All statistical analyses were performed using IBM SPSS Statistics v. 22. Outliers were detected and removed using the boxplot method. An ANOVA was conducted, followed by Duncan’s test (*P* ≤ 0.05) comparing the different groups (denoted by letter). Data was also submitted to Pearson’s correlation analysis and significant correlations were tested through Student’s t-test (*P* ≤ 0.05).

## Results

### *P. glomerata* growth is modulated by osmotic stress and melatonin

**Fig. 1:**
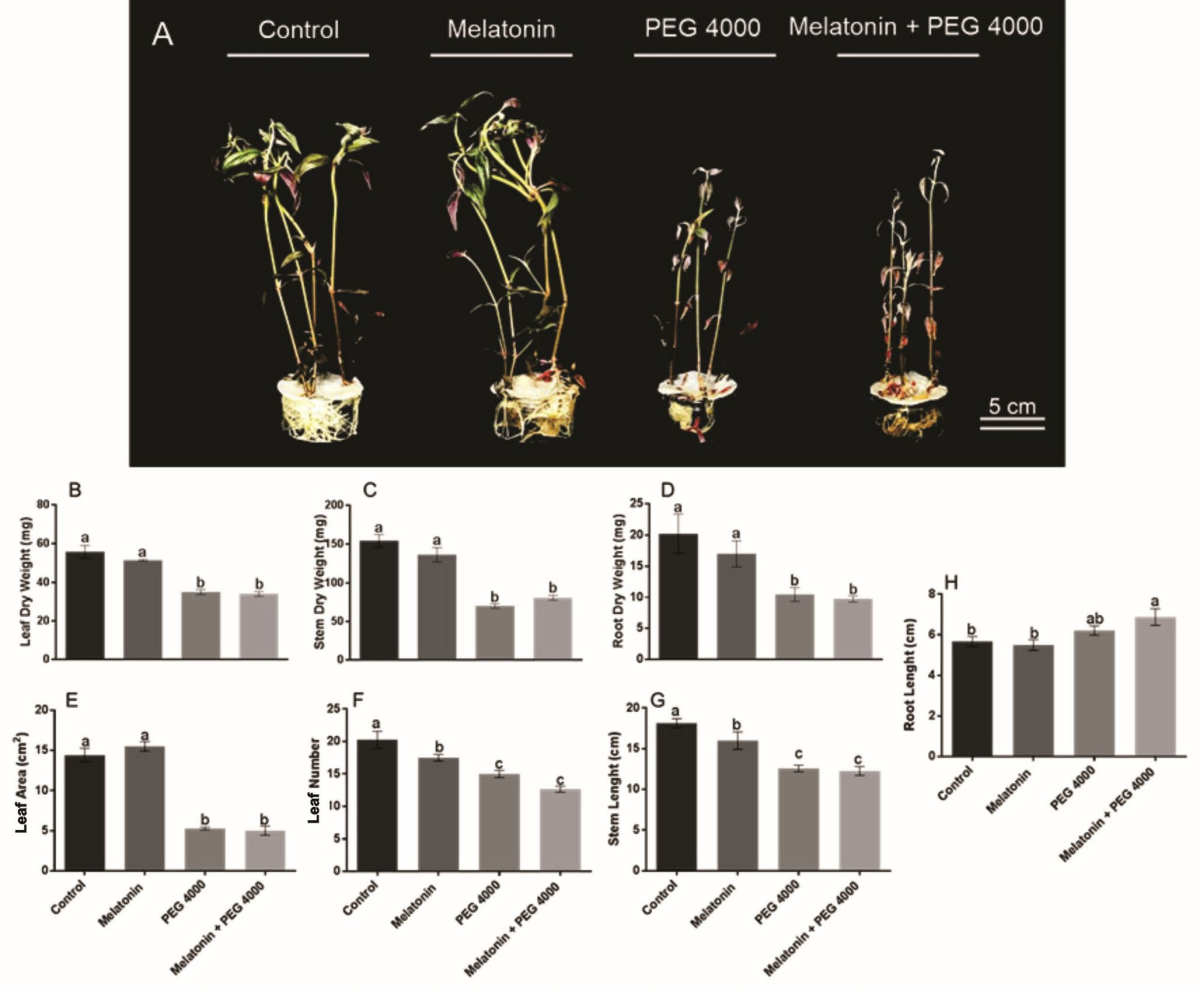
Biometric assessments of *Pfaffia glomerata* grown for 35 days under different treatments: melatonin (100 µM), PEG 4000 (0.25%), and melatonin (100 µM) + PEG 4000 (0.25%). Melatonin and PEG were applied through the culture medium on the tenth day of cultivation: **A** plant stem and leaves architecture, and roots growth pattern; **B** average of leaf dry weight; **C** average stem dry weight; **D** average root dry weight; **E** average leaf area; **F** average number of leaves; **G** average stem length; **H** average root length. Groups sharing the same letters are deemed statistically homogeneous, as established by Duncan’s test (*P* ≤ 0.05). Bars represent the mean ± standard error (*n* = 4 biological replicates from a single experiment)

*P. glomerata* plants treated with PEG, melatonin, and their combination showed distinct growth patterns, as depicted in Fig. 1. Osmotic stress strongly impaired plant growth, with no evident effect of melatonin (Fig. 1a). PEG treatment led to a significant reduction in leaf (37%), stem (55%), and root (48%) dry mass, with no alleviation effect by melatonin (Figs. 1b–d). Leaf area, an important factor related to total photosynthetic capacity, decreased by 65% under PEG treatment compared to control and melatonin-treated plants (Fig. 1e). Interestingly, melatonin-treated plants had 13% fewer leaves than control plants, while the combination of melatonin and PEG resulted in a 37% decrease (Fig. 1f). A similar trend was seen in stem length, with a 32% reduction in plants treated with melatonin + PEG (Fig. 1g). Surprisingly, plants exposed to both melatonin and PEG displayed a 21% increase in root length compared to the control and melatonin groups (Fig. 1h). These results indicate that osmotic stress significantly altered all measured growth parameters in *P. glomerata*, with some parameters possibly intensified by melatonin (e.g., leaf number reduction, stem length reduction, and increased root length).

### Osmotic stress decreases the photosynthetic pigments content in *P. glomerata*

**Fig. 2:**
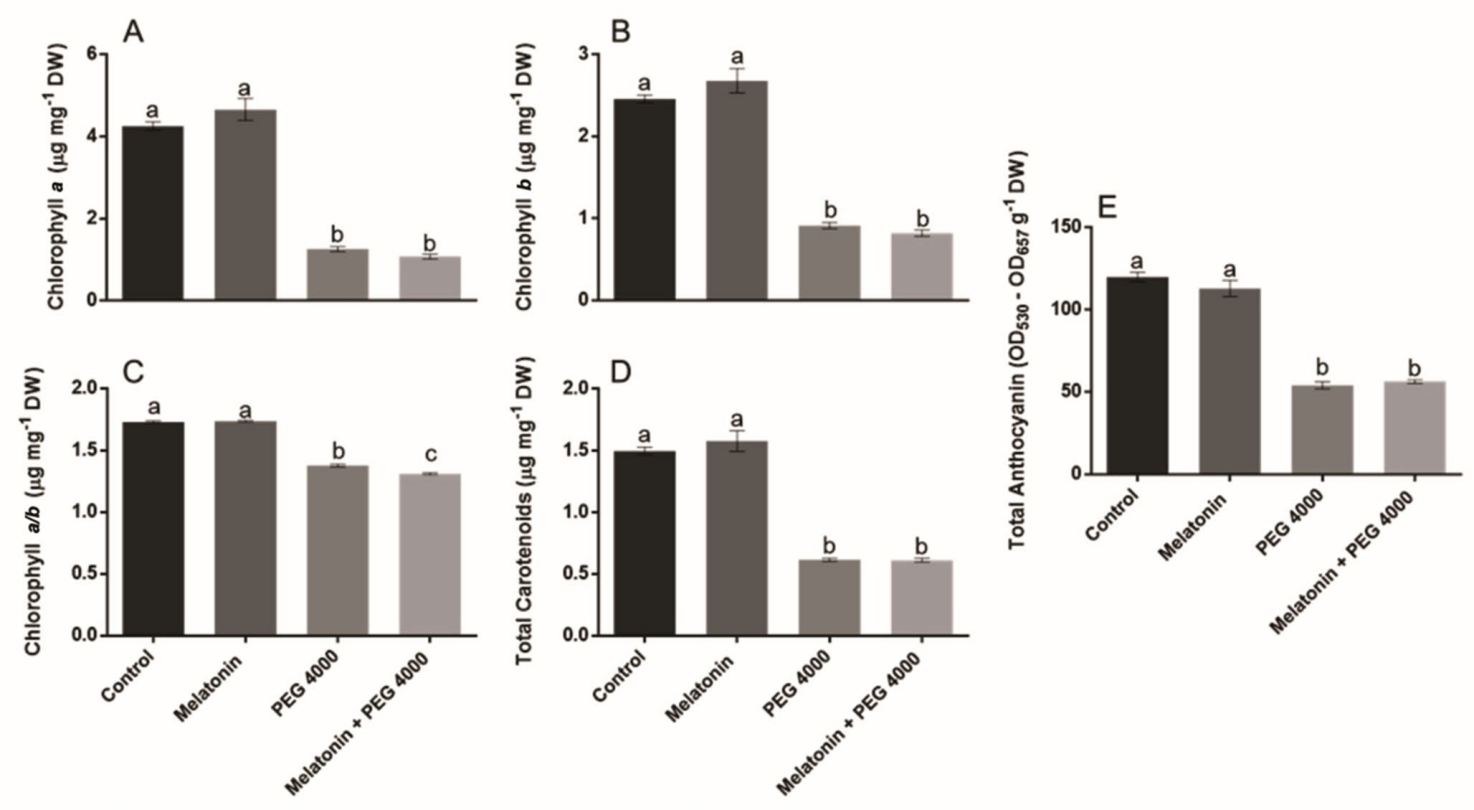
Pigment quantification on *P. glomerata* plants submitted to different treatments: melatonin (100 µM); PEG 4000 (0.25%); and melatonin (100 µM) + PEG 4000 (0.25%): **A** chlorophyll *a* ; **B** chlorophyll *b* **;** **C** chlorophyll *a*/*b* ratio; **D** carotenoids; **E** anthocyanin content. Bars represent the mean ± standard error (*n* = 4 biological replicates from a single experiment). Groups sharing the same letters are deemed statistically homogeneous, as established by Duncan’s test (*P* ≤ 0.05)

Photosynthetic pigment levels often change under osmotic or salt stress conditions (Fortini et al. 2023). In the present study, plants treated with PEG displayed a sharp decline in chlorophyll a (70%) and chlorophyll b (62%), with no significant improvement when melatonin was added (Figs. 2a, b). The chlorophyll *a*/*b* ratio, often an indicator of stress, was reduced by 20% in PEG-treated plants compared to control and melatonin-treated plants. Interestingly, when melatonin was added along with PEG, this reduction was even more pronounced, decreasing by 24% (Fig. 2C). Carotenoid and anthocyanin content also decreased by 58% and 55%, respectively, in PEG-treated plants (Figs. 2D, E).

### The sugar profile is modulated by the presence of PEG and melatonin

**Fig. 3:**
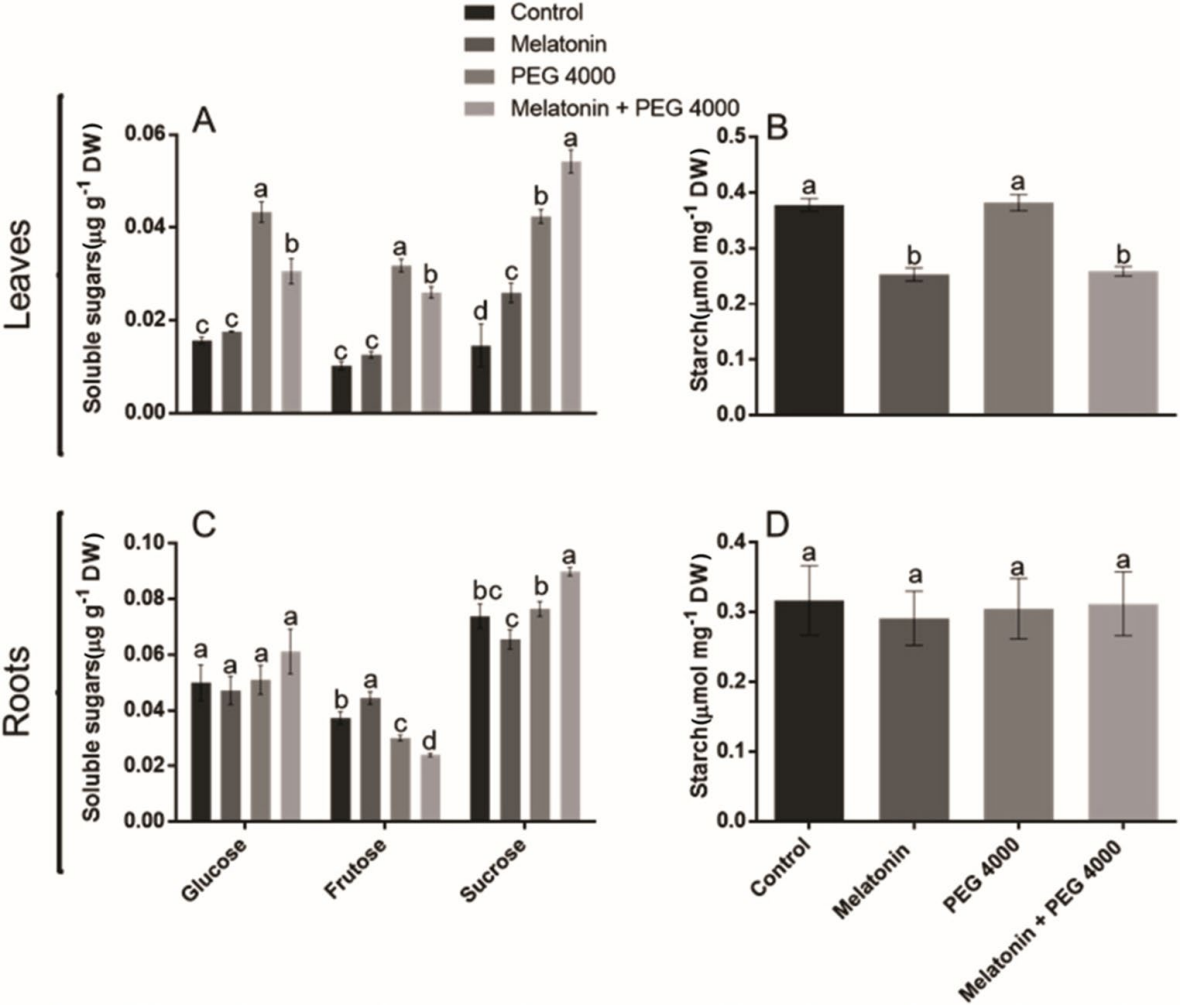
Soluble sugar and starch quantity on leaves and roots of *P. glomerata* plants grown under different treatments: melatonin (100 µM); PEG 4000 (0.25%); and melatonin (100 µM) + PEG 4000 (0.25%): **A** soluble sugars and **B** starch contents on leaves; **C** soluble sugars and **D** starch levels on roots. Bars represent the mean ± standard error (*n* = 4 biological replicates from a single experiment). Groups sharing the same letters are deemed statistically homogeneous, as established by Duncan’s test (*P* ≤ 0.05)

PEG-treated plants had approximately 180% higher glucose content in their leaves compared to the control, while melatonin alone had little effect (Fig. 3A). When melatonin was combined with PEG, glucose levels decreased by about 29% relative to PEG alone. A similar pattern was observed for fructose, with PEG-treated plants showing about a 200% increase compared to the control, whereas the melatonin + PEG treatment resulted in a 17% reduction relative to PEG. Sucrose levels increased across all treatments, being about 67% higher in melatonin-treated plants, 180% higher in PEG-treated plants, and 267% higher in the combination treatment compared to the control (Fig. 3A). Starch levels were approximately 32–34% lower in melatonin and melatonin + PEG treatments than in the control and PEG treatments (Fig. 3B).

In roots, glucose levels remained similar across all treatments (Fig. 3C). However, fructose content increased by about 20% in melatonin-treated plants, while PEG treatment caused a 19% decrease compared to the control, and the combination treatment promoted a 36% reduction (Fig. 3C). Starch content did not vary among treatments in roots (Fig. 3D). Such variations in sugar levels suggest an osmotic adjustment response to PEG 4000-induced stress.

### Amino acids and proline accumulation in *P. glomerata* leaves and roots under PEG and melatonin treatments

**Fig. 4:**
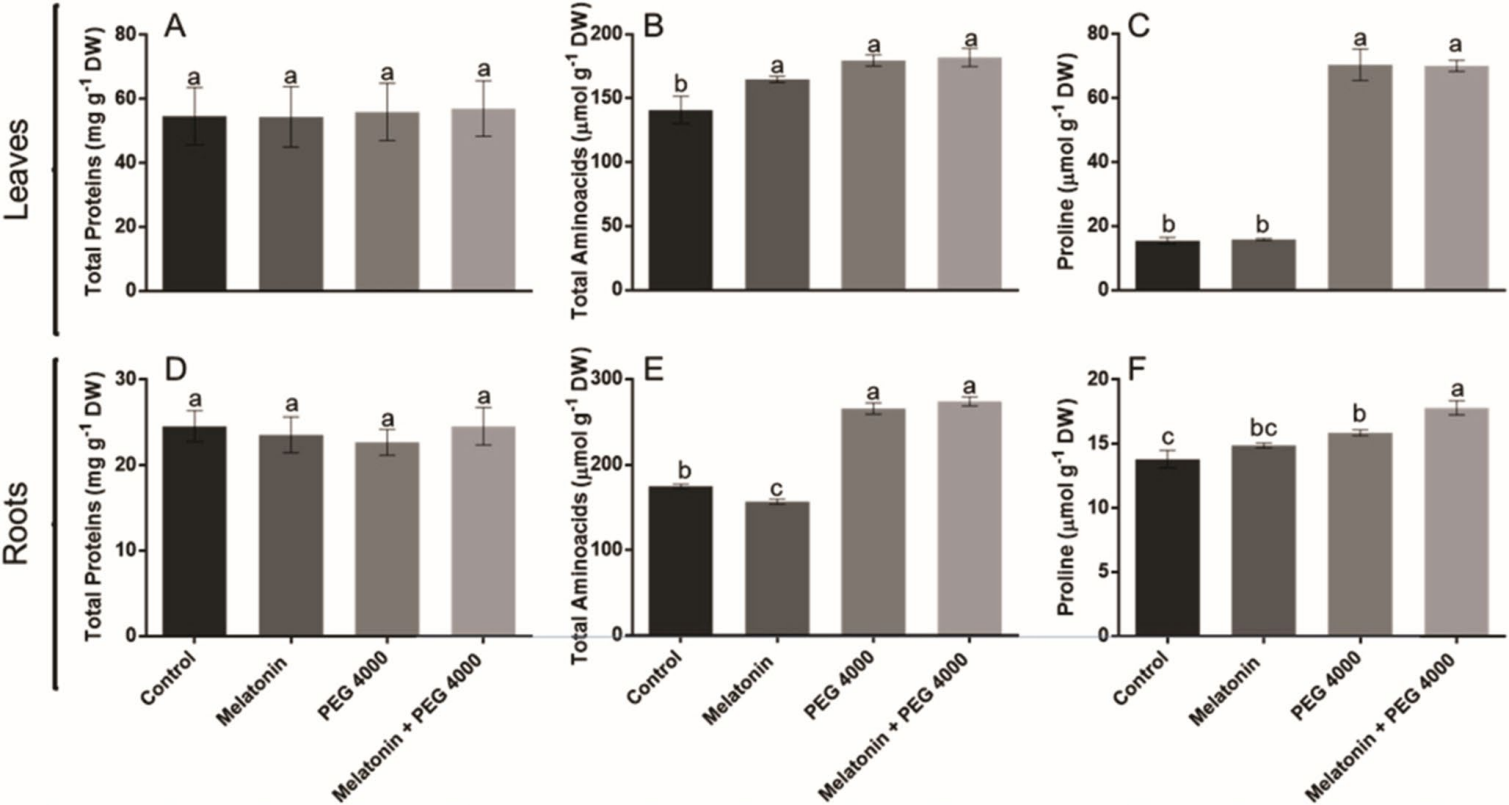
Protein related metabolism in *P. glomerata* plants grown in different treatments: melatonin (100 µM); PEG 4000 (0.25%); and melatonin (100 µM) + PEG 4000 (0.25%): **A** protein, **B** aminoacids, and **C** proline content in leaves; **D** protein, **E** aminoacids, and **F** proline levels in roots. Bars represent the mean ± standard error (*n* = 4 biological replicates from a single experiment). Groups sharing the same letters are deemed statistically homogeneous, as established by Duncan’s test (*P* ≤ 0.05)

To investigate further, we measured total protein, amino acid, and proline content in the leaves and roots of *P. glomerata* plants (Fig. 4). Total protein levels in the leaves and roots did not differ significantly between treatments (Figs. 4A, D). In leaves, plants treated with melatonin, PEG, and their combination showed an increase of approximately 17–29% in amino acid levels compared to the control (Fig. 4B). In roots, PEG and the melatonin + PEG treatments increased amino acid levels by approximately 56% relative to the control (Fig. 4E). Proline levels in leaves treated with PEG and melatonin + PEG were approximately 350% higher than in the control (Fig. 4C). In roots, PEG and the melatonin + PEG treatments increased amino acid levels by approximately 60% compared to the control, while proline levels in melatonin + PEG-treated plants were 28% higher than in the control (Figs. 4E, F). These findings indicate that *P. glomerata* induces osmotic adjustment in response to osmotic stress, with melatonin further contributing to proline accumulation in roots under PEG treatment.

### Osmotic stress and melatonin modulate antioxidant defense in *P. glomerata* plants

**Fig. 5:**
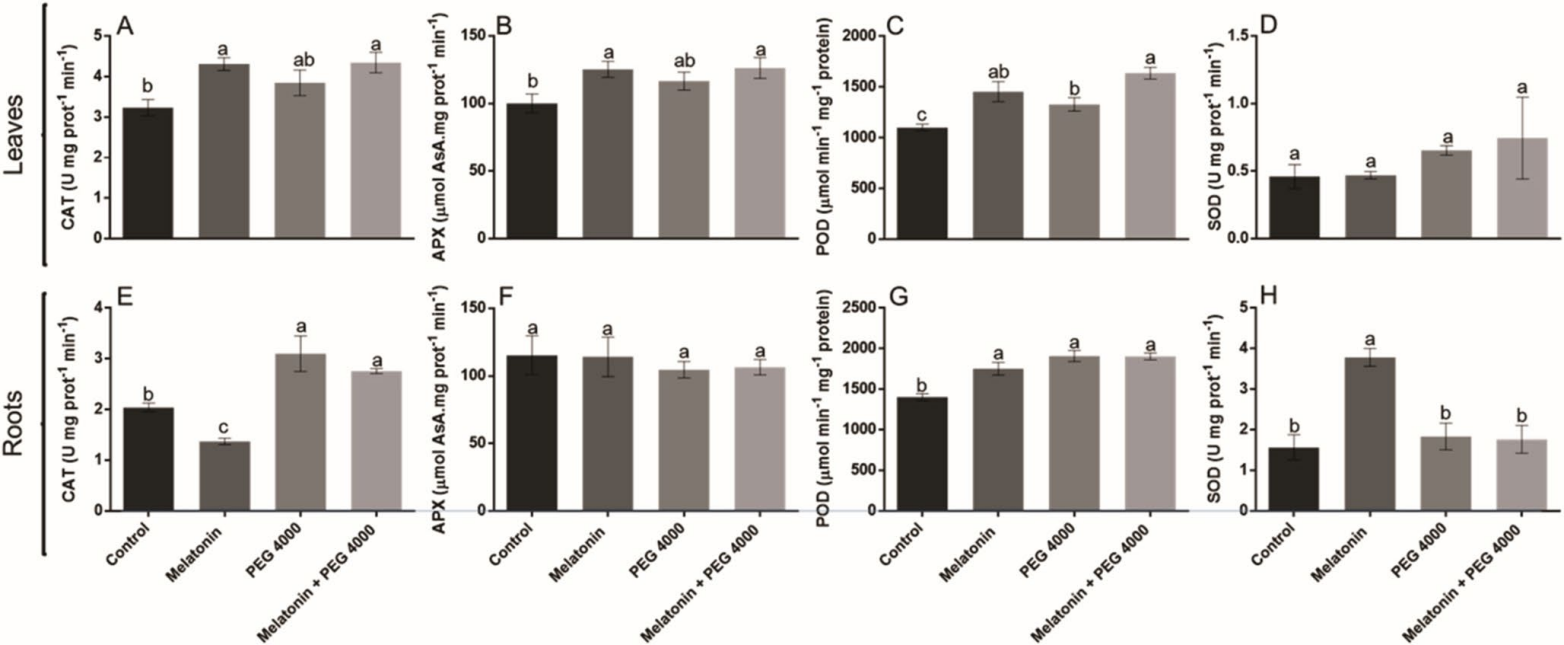
Oxidative metabolism enzymes activity in *P. glomerata* plants submitted to different treatments: melatonin (100 µM); PEG 4000 (0.25%); and melatonin (100 µM) + PEG 4000 (0.25%): **A** catalase (CAT), **B** ascorbateperoxidase (APX), **C **peroxidase oxidoreductase (POD), and **D** superoxide dismutase (SOD) activities in leaves; **E** CAT, **F** APX, **G** POD, and **H** SOD activities in roots. Bars represent the mean ± standard error (*n* = 4 biological replicates from a single experiment). Groups sharing the same letters are deemed statistically homogeneous, as established by Duncan’s test (*P* ≤ 0.05)

To assess antioxidative defense, we measured the activity of key antioxidant enzymes, including catalase (CAT), ascorbate peroxidase (APX), peroxidase (POD), and superoxide dismutase (SOD) in the leaves and roots of *P. glomerata* (Fig. 5). In the leaves, CAT activity was approximately 34% higher in melatonin-treated plants and 37% higher in the combination treatment compared to the control, while PEG-treated plants showed a 19% increase (Fig. 5A). APX activity followed a similar trend, increasing by about 26% in melatonin-treated plants, 16% in PEG-treated plants, and 32% in the combination treatment relative to the control (Fig. 5B). POD activity also increased under all treatments, being about 40% higher in melatonin-treated plants, 30% higher in PEG-treated plants, and 60% higher in the combination treatment compared to the control (Fig. 5C). No significant differences were observed in SOD activity in the leaves (Fig. 5D).

In the roots, CAT activity increased by approximately 55% under PEG treatment and 35% under the combination treatment, while melatonin-treated plants showed a 35% reduction compared to the control (Fig. 5E). POD activity was also higher in treated plants, increasing by about 21% in melatonin-treated plants and 36% in both PEG and combination treatments relative to the control (Fig. 5G). SOD activity was markedly higher in melatonin-treated plants, showing an increase of approximately 140% compared to the control (Fig. 5H). These results indicate that osmotic stress activates antioxidant defenses in *P. glomerata*, particularly increasing POD activity in leaves and CAT and POD activities in roots, while melatonin notably stimulated SOD activity in roots.

### Osmotic stress alters membrane integrity and 20-E levels in *P. glomerata*

**Fig. 6:**
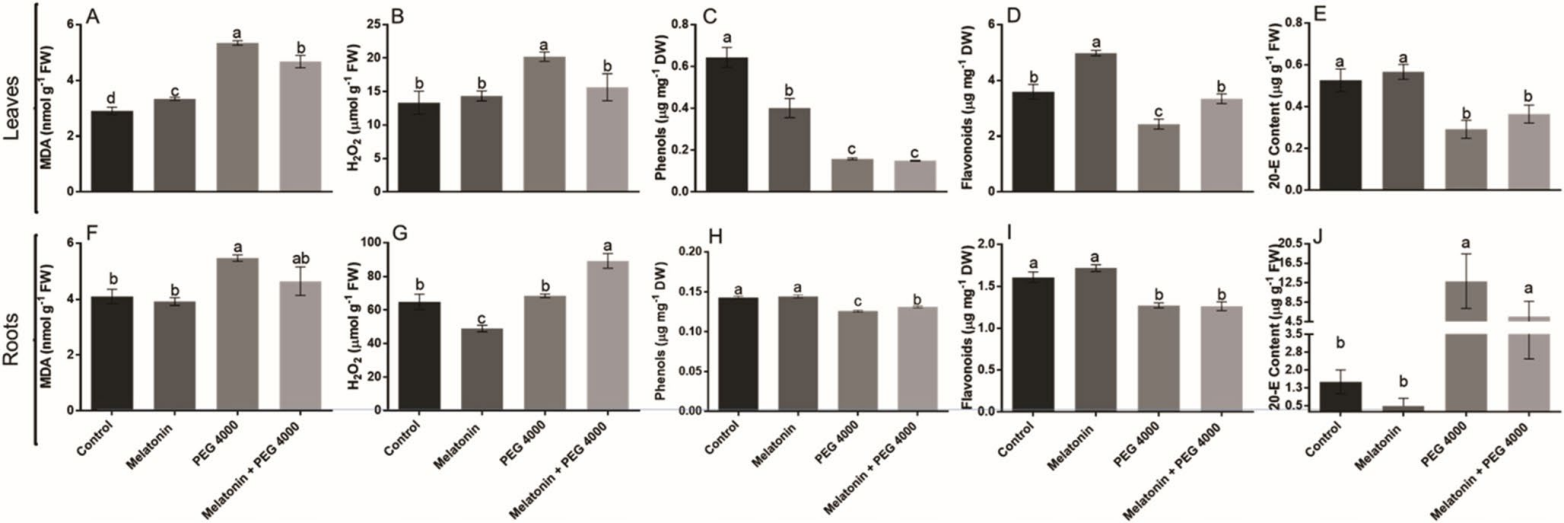
Plant stress-related metabolites on *P. glomerata* leaves and roots grown under different melatonin concentrations: **A** malondialdehyde (MDA), **B** free hydrogen peroxide (H_2_O_2_), **C** phenols and **D** flavonoids levels in leaves; **E** 20-E (20-hydroxyecdysone), **F** MDA, **G** free H_2_O_2_, **H** phenols, **I** flavonoids, and **J** 20-E content in roots. Bars represent the mean ± standard error (*n* = 4 biological replicates from a single experiment). Groups sharing the same letters are deemed statistically homogeneous, as established by Duncan’s test (*P* ≤ 0.05)

Finally, we evaluated the levels of malondialdehyde (MDA), hydrogen peroxide (H₂O₂), phenols, flavonoids, and 20-hydroxyecdysone (20-E) in the leaves and roots (Fig. 6). MDA levels in the leaves of PEG-treated plants were approximately 65% higher than in the control, indicating significant membrane lipid peroxidation (Fig. 6A). Compared to the control, melatonin alone slightly increased MDA content. However, compared to PEG, the melatonin + PEG treatment slightly decreased MDA content, suggesting a membrane-stabilizing effect of melatonin. PEG treatment increased H₂O₂ content by 51% compared to the control, with melatonin reversing this increase (Fig. 6B). Phenolic and flavonoid contents in the leaves were reduced by 32% and 45%, respectively, under PEG treatment compared to the control, while melatonin restored flavonoid levels but had no effect on phenolics (Figs. 6C, D). Interestingly, melatonin alone increased flavonoid content in leaves compared to the control. In the leaves, 20-E levels were lower in plants grown under PEG compared to the control, with melatonin not affecting this reduction (Fig. 6E).

In roots, MDA levels were approximately 40% higher in PEG-treated plants compared to the control, with the combination treatment showing a 15% decrease relative to PEG alone (Fig. 6F). H₂O₂ content in roots was not affected by PEG but decreased under melatonin alone and increased under the melatonin + PEG treatment (Fig. 6G). Phenolic compounds and flavonoids showed similar patterns, with PEG decreasing their contents compared to the control (Figs. 6H, I). Interestingly, melatonin partially reversed the decrease caused by PEG in phenolic content but not in flavonoid levels. PEG strongly increased the accumulation of 20-E (up to 700%) in roots, not differing from the melatonin + PEG combination (Fig. 6J).

These findings indicate that osmotic stress induces marked oxidative damage in *P. glomerata*, particularly in leaves, as reflected by increased ROS levels and membrane lipid peroxidation, while strongly stimulating 20-E accumulation in roots. Melatonin mitigated some of these effects, especially by reducing H₂O₂ levels and membrane damage in leaves, highlighting its potential role in modulating oxidative stress responses under osmotic stress.

### Correlation analysis

**Fig. 7:**
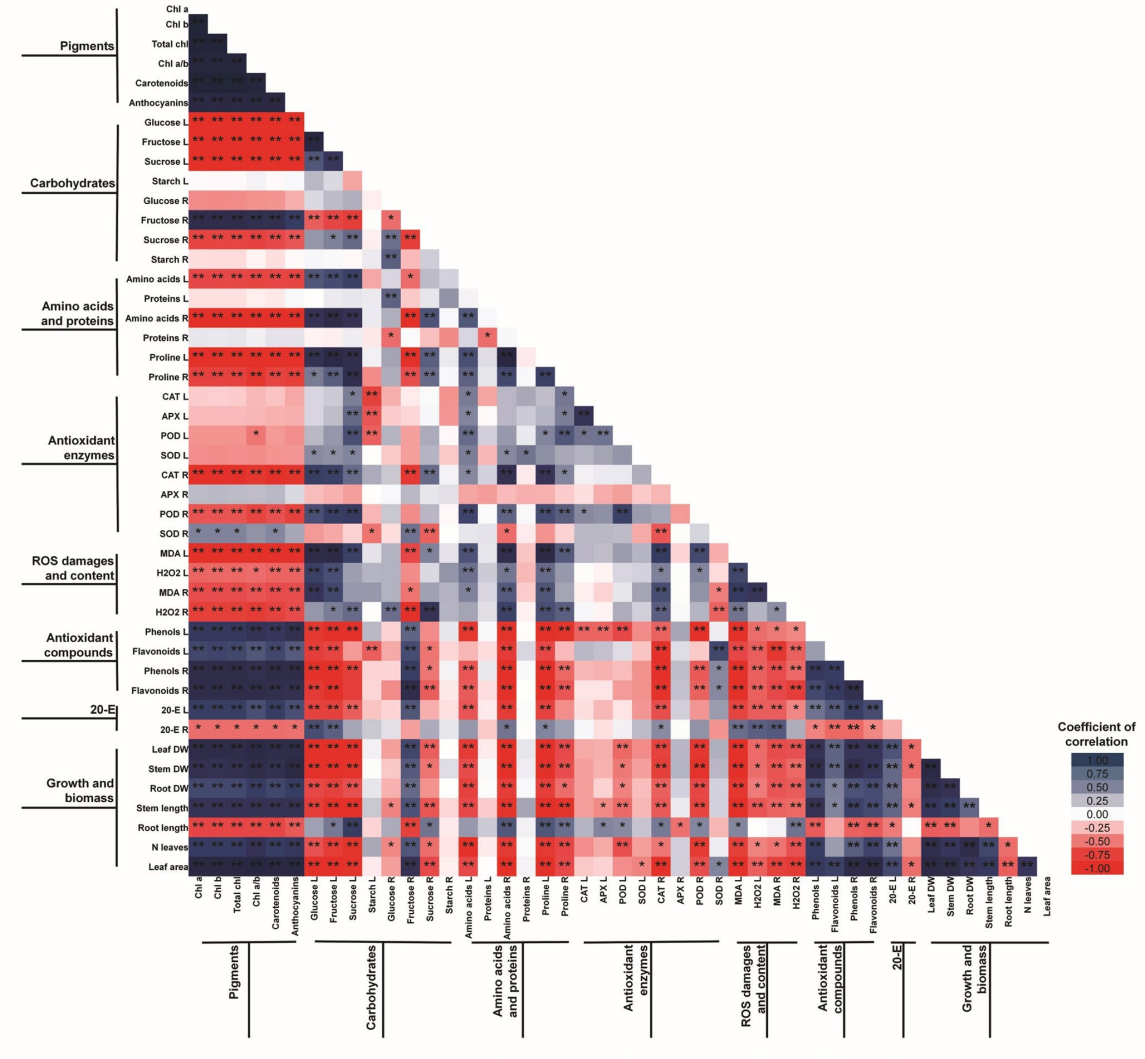
Pearson’s correlation analysis based on biochemical and biometric variables observed on *P. glomerata* leaves and roots grown under PEG-induced osmotic stress and different melatonin concentrations. Asterisks indicate significant correlation at 5% (*) and 1% (**) of probability through student’s t-test. Strong blue color indicates positive and strong red color indicates negative correlations. Chl: chlorophyll; L: leaves; R: roots; DW: dry weight; N leaves: number of leaves

Most growth and biomass variables had strong positive correlations (*P* ≤ 0.01) with pigments and antioxidant compounds, and strong negative correlations with leaf sugars, root sucrose, amino acids, proline, leaf POD, root CAT and POD, MDA, and H₂O₂ (Fig. 7). In contrast, root length was negatively correlated with pigments, root fructose, root APX, and antioxidant compounds, and positively correlated with leaf fructose and sucrose, root sucrose, root amino acids, proline, leaf APX and POD, and root CAT and POD, leaf MDA, and root H₂O₂. Root length also had negative correlations with leaf dry weight, stem dry weight, stem length, number of leaves, and leaf area. Similarly, 20-E contents in leaves and roots showed opposite correlation patterns, with leaf 20-E being positively correlated with pigments, root fructose, antioxidant compounds, and growth variables (except root length), and negatively correlated with leaf sugars, amino acids, leaf proline, root CAT, MDA, H₂O₂, and root length. In contrast, root 20-E showed the opposite correlations and was not correlated with root length (Fig. 7).

As expected, MDA and H₂O₂ levels were negatively correlated with pigments and growth variables (except root length), and positively correlated with leaf sugars, amino acids, and proline. However, MDA and H₂O₂ were not correlated with most antioxidant enzymes, showing positive correlations only with root CAT and POD, and a negative correlation with root SOD. Conversely, MDA and H₂O₂ levels had strong negative correlations with phenolics and flavonoids, suggesting an important role of these antioxidant compounds in ROS scavenging. The negative correlation between MDA and H₂O₂ with leaf 20-E, and the positive association with root 20-E, may indicate that 20-E plays different roles in these organs, potentially contributing to oxidative stress mitigation in leaves but not in roots of *P. glomerata* (Fig. 7).

## Discussion

Turgor pressure is the primary driver of cell expansion; therefore, cell dehydration under osmotic stress directly restricts plant growth (Anjum et al. 2017). Reductions in turgor rapidly activate Ca²⁺- and ROS-mediated signaling cascades that induce ethylene production and arrest leaf expansion within hours (Dubois and Inzé 2020). Subsequently, ABA-mediated stomatal closure suppresses photosynthesis, limiting energy availability and biomass accumulation (Hasanuzzaman et al. 2023). Consistent with this framework, osmotic stress strongly impaired all growth parameters of *P. glomerata*, except root length. Melatonin did not reverse growth inhibition in most traits but slightly increased root length, indicating partial stress alleviation.

The increase in root length observed under the melatonin + PEG treatment may be associated with modulation of auxin-related signaling pathways. Melatonin has been reported to influence root system architecture through mechanisms that partially converge with auxin signaling, including the regulation of root meristem activity, cell elongation, and auxin transport, ultimately affecting root growth dynamics under stress conditions (Liang et al. 2026). In our study, the positive association between root elongation and the accumulation of compatible solutes such as sucrose, amino acids, and proline further suggests that root growth under osmotic stress was accompanied by metabolic adjustments supporting osmotic regulation. Given that hydrotropism is a typical response to osmotic stress (Miyazawa and Takahashi 2020), these results suggest that melatonin may modulate root hydrotropism in *P. glomerata*, enhancing root exploration of water-limited environments. This interpretation is supported by strong negative correlations between root length and shoot growth parameters, revealing a clear trade-off in resource allocation. Increased root elongation reflects stress-induced plasticity that prioritizes water foraging over carbon investment in aerial tissues.

Root length was positively correlated with proline, sucrose, and total amino acid contents in roots, indicating that root elongation under osmotic stress is closely associated with the accumulation of compatible solutes. This response agrees with findings in *Arabidopsis*, where proline regulates root meristem size and promotes root growth (Biancucci et al. 2015). However, root elongation was accompanied by increased oxidative damage, as evidenced by negative correlations with phenolics and flavonoids and positive correlations with MDA and H₂O₂. The positive association between proline content, antioxidant enzymes, and H₂O₂ further indicates that proline accumulation primarily supported osmotic adjustment rather than oxidative detoxification. Indeed, proline metabolism has been shown to modulate H₂O₂ levels, which in turn regulate root growth (Bauduin et al. 2022).

Osmotic stress also impaired photosynthetic capacity in *P. glomerata*, as indicated by marked reductions in photosynthetic pigments, an effect not alleviated by melatonin. Chlorophyll degradation under osmotic stress is driven by enhanced activity of pheophorbide-a-oxygenase, pheophytinase, and chlorophyllase (Zahra et al. 2023), linking pigment loss to growth inhibition. Similar reductions in chlorophyll content have been reported in salt-stressed *P. glomerata* (Fortini et al. 2023). Strong positive correlations between pigment levels and growth parameters, together with negative correlations with MDA and H₂O₂, indicate that oxidative stress contributed to pigment degradation and constrained biomass production.

Melatonin has been proposed to regulate carbon allocation by modulating carbohydrate metabolism, including the partitioning of Calvin cycle products and the balance between starch and sucrose pools (Arnao et al. 2021). In some plant systems, exogenous melatonin alleviates stress-induced carbon limitations by sustaining photosynthesis and carbohydrate homeostasis, as reported under low-light stress in tobacco seedlings (Xu et al. 2025). However, these responses appear to be strongly dependent on the nature and intensity of the stress. In the present study, under PEG-induced osmotic stress, melatonin did not prevent the decline in photosynthetic pigments and was unable to restore photosynthetic performance in *P. glomerata*. The reduction in chlorophyll content and the strong inhibition of shoot growth indicate that osmotic stress imposed severe constraints on the photosynthetic apparatus. Thus, although melatonin enhanced metabolic adjustments associated with stress mitigation, these effects were insufficient to sustain carbon assimilation under prolonged osmotic stress.

Under osmotic stress, plants commonly induce osmotic adjustment through the accumulation of compatible solutes to maintain cellular hydration and sustain water uptake (Kaur et al. 2024). Extreme examples include *Craterostigma plantagineum*, which survives complete dehydration through massive sucrose accumulation (Norwood et al. 2000; Xu et al. 2021). Amino acids, particularly proline, are also central to osmotic adjustment and are often considered biochemical markers of severe stress (Ghosh et al. 2021; Spormann et al. 2023). Early activation of genes involved in proline and branched-chain amino acid biosynthesis further links amino acid metabolism to abiotic stress responses (Buffagni et al. 2020; Trovato et al. 2021), while proline can enhance nutrient uptake under osmotic stress (Ali et al. 2008; Hayat et al. 2012).

In the present study, osmotic stress increased sucrose, total amino acids, and proline levels in both leaves and roots. Melatonin further enhanced sucrose accumulation in both organs and increased proline levels in roots, reinforcing its role in promoting osmotic adjustment, as widely reported across plant species (Dzinyela et al. 2024). These compatible solutes may also contribute to ROS buffering and signaling processes (Hennion et al. 2019; Salmon et al. 2020), helping to stabilize cellular redox balance and maintain turgor under osmotic stress.

Despite these benefits, osmolyte accumulation entails substantial metabolic costs. Proline acts as a metabolic hub that redirects resources from growth toward stress responses under drought and salinity (Alvarez et al. 2022). In *Brassica napus*, proline metabolism has been linked to source–sink regulation and senescence processes (Dellero et al. 2020a, b), which may explain the reduced leaf number observed in PEG-stressed *P. glomerata*. The strong negative correlations between proline content and growth parameters further highlight the trade-off between growth and stress adaptation, illustrating how resource allocation shifts from biomass production toward survival-oriented metabolic adjustments under osmotic stress.

The persistence of growth inhibition under osmotic stress, even with melatonin supplementation, reflects coordinated metabolic reprogramming rather than failure of a single protective mechanism. Melatonin acted as a context-dependent regulator that enhanced stress adjustment without restoring growth, consistent with previous reports (Gao et al. 2022; Rezaei et al. 2025). Reduced chlorophyll content limited photosynthetic carbon assimilation, while elevated ROS imposed additional metabolic costs for cellular maintenance. Although melatonin mitigated oxidative damage, it did not fully restore pigment levels or carbon balance, explaining the absence of growth recovery. Osmotic adjustment alone was insufficient to restore turgor-driven cell expansion under sustained stress. Concurrently, increased 20-hydroxyecdysone (20-E) levels likely reflect stress-induced metabolic reprogramming that favors acclimation over biomass production, as reported for other medicinal plants in which melatonin primarily regulates carbon and secondary metabolism allocation (Mahmood et al. 2025).

Osmotic stress induced marked oxidative damage in *P. glomerata*, particularly membrane damage associated with elevated MDA and H₂O₂. Besides affecting membrane integrity, lipid peroxidation products such as malondialdehyde can also interact with proteins and nucleic acids, potentially altering enzyme activity and inducing DNA damage. Melatonin partially reduced H₂O₂ levels and membrane damage in leaves but not in roots. This protective effect in leaves was accompanied by increased flavonoid content and peroxidase activity, responses absent in roots. Flavonoids are key non-enzymatic antioxidants involved in ROS scavenging and drought tolerance (Shomali et al. 2022), while peroxidases detoxify H₂O₂ using phenolics as substrates (Czégény and Racz 2023). Despite reduced phenolic content under osmotic stress, the melatonin-induced enhancement of flavonoids and peroxidase activity indicates a leaf-specific antioxidant strategy. In contrast, roots showed increased H₂O₂ under melatonin treatment, reinforcing that proline accumulation in roots primarily supported osmotic regulation rather than ROS scavenging, as confirmed by positive correlations with oxidative damage markers. This reinforces that root responses prioritize osmotic regulation rather than redox homeostasis.

Several correlations initially appeared counterintuitive but provide important biological insights. The positive association between proline and oxidative damage in roots indicates that, under severe osmotic stress, proline functions mainly as an osmotic regulator rather than an effective antioxidant. Similarly, the association of increased root 20-E with oxidative damage suggests that its accumulation reflects stress severity rather than conferring direct protection. These patterns underscore that stress-induced metabolic adjustments do not necessarily translate into growth recovery or oxidative mitigation. Importantly, although root 20-E levels increased under PEG-induced osmotic stress, this increase did not coincide with reduced membrane damage, as MDA levels in roots were not decreased in the absence of melatonin (Fig. 6f). The reduction in MDA observed under the melatonin + PEG treatment therefore cannot be attributed to 20-E accumulation, indicating that melatonin-mediated redox protection operates independently of 20-E dynamics in roots.

Leaf and root responses to melatonin differed markedly, reflecting tissue-specific priorities. Leaves benefited from reinforced redox homeostasis and membrane integrity, supporting photosynthetic function, whereas roots prioritized osmotic adjustment and exploratory growth. As roots are the primary site of osmotic stress perception and the main site of 20-E biosynthesis in *P. glomerata*, they favored solute accumulation and elongation over oxidative detoxification. This tissue-specific modulation highlights the role of melatonin in reallocating stress responses rather than uniformly enhancing protection.

Abiotic stresses such as salinity have been shown to increase 20-E production in *P. glomerata* (Felipe et al. 2019), although responses depend on stress severity and growth conditions (Fortini et al. 2023). In this study, PEG-induced osmotic stress increased 20-E in roots but decreased it in leaves. Opposite correlation patterns between 20-E and growth or oxidative damage further support tissue-specific regulation. In leaves, positive associations between 20-E and growth, together with negative correlations with oxidative damage, suggest a protective role linked to redox balance. In roots, positive correlations with MDA and H₂O₂ and negative associations with growth indicate that 20-E accumulation accompanies stress intensity rather than stress mitigation. Given that roots are the primary site of 20-E production and long-term accumulation in *P. glomerata* (Festucci-Buselli et al. 2008; Ribeiro et al. 2024), this response is expected. Moreover, 20-E accumulation appears specific to osmotic stress, as photoperiod and light quality did not alter its levels under comparable stress conditions (Fortini et al. 2020; Silva et al. 2020, 2021).

The increase in root 20-E under osmotic stress occurred independently of melatonin, indicating that it represents a stress-driven response rather than a downstream effect of melatonin signaling. Notably, elevated 20-E did not reduce membrane damage, reinforcing that it does not act primarily as an antioxidant in roots. Instead, 20-E likely contributes to adaptive developmental or metabolic adjustments rather than direct ROS scavenging. Although 20-E has been reported to enhance antioxidant capacity under abiotic stress in other systems (Li et al. 2018; Arif et al. 2022), this function appears tissue-dependent in *P. glomerata*. While leaf 20-E correlated negatively with oxidative damage and positively with antioxidant traits, root 20-E showed the opposite pattern and was associated with proline metabolism. Further studies are required to clarify these tissue-specific roles.

The potential interactions between 20-E and brassinosteroids warrant further investigation. Both steroid classes contribute to membrane stabilization and stress signaling (Bartwal et al. 2013; Rogowska and Szakiel 2020; Du et al. 2022; Manghwar et al. 2022). Sterol metabolism is responsive to drought, as shown in rice cultivars that adjust sterol profiles to protect membranes (Kumar et al. 2018). Enzymes such as C5SD are central to these responses, and their manipulation enhances drought tolerance through improved ROS scavenging (Zhang et al. 2019), consistent with increased antioxidant enzyme activities observed in *P. glomerata* leaves. Brassinosteroid signaling has also been linked to proline accumulation and root hydrotropism (Jardim-Messeder et al. 2025). Melatonin can act synergistically with brassinosteroids and H₂O₂ to enhance osmotic adjustment, antioxidant defenses, and root growth under drought and salinity (Fu et al. 2022; Yusuf et al. 2025), which may explain the increased root length observed here. Future studies integrating melatonin, brassinosteroid signaling, and tissue-specific 20-E dynamics will be essential to fully elucidate stress acclimation mechanisms in *P. glomerata*.

Collectively, these findings demonstrate that osmotic stress triggers a coordinated metabolic reprogramming in *Pfaffia glomerata*, in which growth limitation results from turgor loss, impaired photosynthetic capacity, and increased metabolic costs associated with osmotic adjustment and oxidative stress. Melatonin acts as a context-dependent regulator that modulates stress adjustment and resource allocation rather than restoring growth, reinforcing antioxidant protection in leaves while favoring osmotic adjustment and root elongation. In parallel, 20-hydroxyecdysone exhibits a marked tissue-specific response, being associated with protective and redox-related functions in leaves, but reflecting stress intensity and adaptive reprogramming in roots rather than direct antioxidant action. Together, these results advance the understanding of how primary metabolism, stress signaling, and steroidal metabolism are differentially integrated under osmotic stress in a medicinal plant system.

## Conclusion

Our study demonstrates that PEG-induced osmotic stress markedly affects growth, photosynthetic pigments, osmolyte accumulation, and oxidative stress markers in *Pfaffia glomerata*, with melatonin only partially alleviating these effects. Rather than restoring growth, melatonin promoted tissue-specific stress adjustment by enhancing antioxidant protection in leaves and osmotic regulation and root elongation under osmotic stress, contributing to plant survival and establishment under water-limiting conditions. Importantly, the increase in 20-hydroxyecdysone was primarily driven by osmotic stress itself and occurred independently of melatonin, indicating that controlled stress conditions may play a central role in secondary metabolite accumulation. From an applied perspective, these findings suggest that melatonin may be used as a complementary tool to improve stress resilience without compromising metabolite production, although its effects are context-dependent. Therefore, optimization of application timing, dosage, and stress intensity will be essential to maximize benefits while avoiding unnecessary metabolic costs in cultivation systems.

## Data Availability

The authors confirm that the data supporting the findings of this study are available within the article.
